# Early Cytoskeletal Protein Modifications Precede Overt Structural Degeneration in the DBA/2J Mouse Model of Glaucoma

**DOI:** 10.3389/fnins.2016.00494

**Published:** 2016-11-03

**Authors:** Gina N. Wilson, Matthew A. Smith, Denise M. Inman, Christine M. Dengler-Crish, Samuel D. Crish

**Affiliations:** ^1^Department of Pharmaceutical Sciences, Northeast Ohio Medical UniversityRootstown, OH, USA; ^2^School of Biomedical Sciences, Kent State UniversityKent, OH, USA; ^3^Integrated Pharmaceutical Medicine Program, Northeast Ohio Medical UniversityRootstown, OH, USA

**Keywords:** glaucoma, cytoskeleton, neurofilament, spectrin, tau, phosphorylation, axonal transport, amyloid-beta

## Abstract

Axonal transport deficits precede structural loss in glaucoma and other neurodegenerations. Impairments in structural support, including modified cytoskeletal proteins, and microtubule-destabilizing elements, could be initiating factors in glaucoma pathogenesis. We investigated the time course of changes in protein levels and post-translational modifications in the DBA/2J mouse model of glaucoma. Using anterograde tract tracing of the retinal projection, we assessed major cytoskeletal and transported elements as a function of transport integrity in different stages of pathological progression. Using capillary-based electrophoresis, single- and multiplex immunosorbent assays, and immunofluorescence, we quantified hyperphosphorylated neurofilament-heavy chain, phosphorylated tau (ptau), calpain-mediated spectrin breakdown product (145/150 kDa), β–tubulin, and amyloid-β_42_ proteins based on age and transport outcome to the superior colliculus (SC; the main retinal target in mice). Phosphorylated neurofilament-heavy chain (pNF-H) was elevated within the optic nerve (ON) and SC of 8–10 month-old DBA/2J mice, but was not evident in the retina until 12–15 months, suggesting that cytoskeletal modifications first appear in the distal retinal projection. As expected, higher pNF-H levels in the SC and retina were correlated with axonal transport deficits. Elevations in hyperphosphorylated tau (ptau) occurred in ON and SC between 3 and 8 month of age while retinal ptau accumulations occurred at 12–15 months in DBA/2J mice. *In vitro* co-immunoprecipitation experiments suggested increased affinity of ptau for the retrograde motor complex protein dynactin. We observed a transport-related decrease of β-tubulin in ON of 10–12 month-old DBA/2J mice, suggesting destabilized microtubule array. Elevations in calpain-mediated spectrin breakdown product were seen in ON and SC at the earliest age examined, well before axonal transport loss is evident. Finally, transport-independent elevations of amyloid-β_42_, unlike pNF-H or ptau, occurred first in the retina of DBA/2J mice, and then progressed to SC. These data demonstrate distal-to-proximal progression of cytoskeletal modifications in the progression of glaucoma, with many of these changes occurring prior to complete loss of functional transport and axon degeneration. The earliest changes, such as elevated spectrin breakdown and amyloid-β levels, may make retinal ganglion cells susceptible to future stressors. As such, targeting modification of the axonal cytoskeleton in glaucoma may provide unique opportunities to slow disease progression.

## Introduction

Glaucoma is a major cause of irreversible blindness, predicted to afflict nearly 112 million people worldwide by the year 2020 (Tham et al., 2014). While age and sensitivity to elevated intraocular pressure (IOP) are two major risk factors, ultimately it is the dysfunction and degeneration of retinal ganglion cells and their axons that lead to permanent vision loss in glaucoma (Quigley, 1999; Almasieh et al., 2012). As in many other chronic neurodegenerations, axonopathy is a component of early glaucoma pathogenesis (Libby et al., 2005; McKinnon et al., 2009; Crish et al., 2010; Dengler-Crish et al., 2014; Cooper et al., 2016). Furthermore, axonopathy is compartmentalized, with axonal transport deficits, morphological changes, and eventual axon loss initially manifested in the distal segments of retinal ganglion cell (RGC) axons (Libby et al., 2005; Schlamp et al., 2006; Crish et al., 2010, 2013; Dengler-Crish et al., 2014; Cooper et al., 2016). Functional axonal transport deficits are observed before structural loss—wherein lies a therapeutic window separating the earliest elements of dysfunction in this disease from stages of irrevocable neural loss (Crish et al., 2010; Sunico et al., 2011; Dengler-Crish et al., 2014).

Axonal transport blockade has been linked to aberrant cytoskeletal organization or breakdown (Coleman, 2005; Shea and Chan, 2008), and cytoskeletal proteins have been historically used as pathological markers in glaucoma (Soto et al., 2011). Recent work has redirected attention to these elements as potential mechanisms for disease progression (Balaratnasingam et al., 2008; Kang et al., 2014; Cooper et al., 2016). In the DBA/2J mouse, a well-characterized model that develops progressive glaucomatous pathology as a function of age (John et al., 1998), optic nerves (ON) from pathologically-advanced mice show striking evidence of large swellings (spheroids) exhibiting accumulated cargo and disorganized, highly phosphorylated neurofilaments (Crish et al., 2010). However, the nature and time course of cytoskeletal abnormalities within the axon have not been clearly defined; these changes may explain the underlying mechanics of early axonal transport deficits characteristic of glaucoma.

The axonal cytoskeleton is dynamic, and arrangement of its components is tightly regulated to produce the proper organization essential for normal structure and function of the axon (Köpke et al., 1993; Nicolas et al., 2002; Ackerley et al., 2003; Jung et al., 2005; Song et al., 2013; Xu et al., 2013; Wortman et al., 2014). Axonopathy often begins with post-translational modifications of proteins involved in axon structure and function (Petzold et al., 2008). Aberrations in several cytoskeletal proteins, such as neurofilaments, spectrin, microtubules, and tau, have been implicated in the pathogenesis of neurodegenerative diseases, including glaucoma (Braak et al., 1994; Schultz et al., 1997; Ahlijanian et al., 2000; Fischer et al., 2004; Tahzib et al., 2004; Cuchillo-Ibanez et al., 2008; Petzold et al., 2008; Chidlow et al., 2011; Haines et al., 2011; Ito et al., 2012; Yan and Jeromin, 2012). Amyloid-β (Aβ) has been a biomarker of neurodegeneration most commonly associated with Alzheimer's disease (AD), but is present in other pathological conditions, including glaucoma (McKinnon, 2003). Although it is not directly involved in the structural maintenance of the cytoskeleton, Aβ may play an active role in axonal transport deficits. Elevations in Aβ protein fibrils and oligomers coincide with elevated intracellular calcium concentrations (Kawahara and Kuroda, 2000), and oligomeric Aβ has been associated with inhibition of fast axonal transport (Morfini et al., 2009). Evidence of Aβ in animal models of glaucoma as well as in human glaucomatous eyes (McKinnon, 2003; Ito et al., 2012) further asserts the necessity of considering the role of such AD pathophysiological elements in the context of glaucoma.

In the current study, we assessed the relationship between cytoskeletal changes, axonal transport deficits, and primary risk factors (i.e., age) in glaucoma. Using a combination of neuronal tract tracing, immunofluorescence, and advanced protein quantitation techniques, we examined levels of extremely hyperphosphorylated (i.e., superphosphorylated) neurofilament (pNF-H), phosphorylated tau (ptau-231), spectrin breakdown product (SBDP 145/150; cleaved αII-spectrin), β-tubulin, and Aβ_42_ throughout the retinal projection of DBA/2J mice as a function of age and extent of axonal transport deficits. Overall, our data support that initial cytoskeletal changes in the distal retinal projection occur before large-scale anterograde transport loss and subsequent axon degeneration, emphasizing the importance of these elements as potential targets for early intervention in the disease.

## Methods

### DBA/2J mouse model of glaucoma

Eighty-three mixed-sex (30 male, 53 female) DBA/2J and DBA/2J-*Gpnmb*^+^ mice of different ages were used for protein quantification and immunofluorescence studies. The DBA/2J mouse has two loss of function mutations that produce iris atrophy, resulting in age-related elevation of IOP and progressive degeneration of visual structures that mimic human glaucoma (John et al., 1998; Burroughs et al., 2011). DBA/2J-*Gpnmb*^+^ mice (D2G) have the same genetic background as DBA/2J mice; however, they express a functioning wild-type *Gpnmb*^+^ allele that prevents development of elevated IOP or glaucomatous pathology (Howell et al., 2007). For our experimental staging in the DBA/2J strain, we used 3–5 month old mice (D3-5) representing pre-glaucomatous ages, 8–10 month old mice (D8-10) representing early glaucomatous pathology where anterograde transport deficits and mild axonopathy are evident, and 12–15 month old mice (D12-15) representing increasing transport deficits, axonopathy, and RGC soma loss characteristic of late glaucomatous pathology. Staging of DBA/2J glaucomatous mice was consistent with previous work published by our lab as well as others and was based on anterograde and retrograde transport loss as well as eventual RGC loss in this model (John et al., 1998; Howell et al., 2007; Buckingham et al., 2008; Crish et al., 2010; Dengler-Crish et al., 2014; Wilson et al., 2015). For control comparisons, we used 3–5 and 12–15 month old D2G mice to represent ages targeted for pre-glaucomatous and late glaucomatous time points in the DBA/2J strain (G3-5 and G12-15 respectively). Table 1 describes experimental and control group nomenclature and provides specific sample sizes. All mice were originally obtained from The Jackson Laboratory (Bar Harbor, ME, USA) and were housed and aged under the same conditions in the Comparative Medicine Unit at Northeast Ohio Medical University. Mice were maintained on a 12-h light/dark cycle with standard rodent chow available *ad libitum*. All experimental procedures were conducted in accordance with the guidelines of the Northeast Ohio Medical University Institutional Animal Care and Use Committee (IACUC). All protocols for animal use were approved by this IACUC prior to initiation of the study.

**Table 1:** **Group nomenclature**.

**Strain**	**Age**	**Group nomenclature**	**Projections analyzed**					
			**pNF-H**	**Aβ42**	**ptau-231**	**Total tau**	**SBDP**	**β-tubulin**
**D2G**	3–5	D2G^*^	8	12	4	4	–	–
**D2G**	12–15		8	6	4	4	4	4
**DBA/2J**	3–5	D3–5	10	14	9	9	4	4
**DBA/2J**	8–10	D8–10	18	14	6	6	7^**^	7^**^
**DBA/2J**	12–15	D12–15	16	18	6	6		

* *Single asterisks denotes that both young and old controls were collapsed into a single group, when possible, to simplify analyses*.

** *Double asterisks denotes that a new combination of ages was used for the SBDP and β-tubulin analyses. An overarching “pathological” group consisting of animals aged 10–12 months was used here*.

### Anterograde tracing

Anterograde tract tracing methods were used to assay anterograde transport integrity between each retina to the corresponding contralateral superior colliculus (Crish et al., 2010). Mice were anesthetized with 2.5% isoflurane and placed prone in a stereotaxic device (Stoelting, Wood Dale, IL). Cholera toxin B-subunit conjugated to Alexa Fluor 488 (CTB) was injected into the vitreous chamber of both eyes (1.5 μl of 0.1% CTB in sterile physiological saline per eye; Life Technologies: Grand Island, NY) using a 33-gage needle attached to a 25 μl Hamilton syringe. Mice were then allowed to recover and were returned to their home cages. Forty-eight hours later, mice were sacrificed by either transcardial perfusion with 4% paraformaldehyde for immunofluorescence assays or by decapitation under 5% isoflurane anesthesia to collect fresh tissue for protein quantitation procedures.

### Tissue collection

Eyes, brain, and ONs were harvested from mice perfused for immunofluorescence assays; brains were post-fixed overnight while eyes and ONs were post-fixed for 2 h. Tissue was transferred to 20% sucrose in phosphate buffered saline (PBS) prior to sectioning. Brains were sectioned coronally through the rostral-caudal extent of the SC at 50 μm on a freezing-sliding microtome; ONs were sliced longitudinally at 20 μm. Retinas were dissected from eyes.

In mice sacrificed for protein quantitation, retina, ONs, SC, and cerebellum (as a control structure) were collected and immediately frozen on dry ice, following the microdissection procedure previously described in Wilson et al. (2015). Whole-mount SC imaging was done with a Zeiss AxioZoom V16 epifluorescent microscope equipped with a digital high-resolution camera (AxioCam MRm Rev.3; Zeiss: Jena, Germany). Under 16.2x magnification, microdissection of the SC was performed to collect transport-intact (CTB-positive) and transport-absent (CTB-negative) regions which were analyzed separately in order to parse out the relationship between transport outcome and protein levels. Retinas were flattened and examined to determine success of the tracer injection as described in Dengler-Crish et al. (2014). Tissue was stored frozen at −80°C until use.

### Sandwich ELISAs for pNF-H and Aβ_42_

Enzyme-linked immunosorbent assays (ELISA) were used to quantify pNF-H (ELISA-pNFH-V1: EnCor Biotechnology Inc.; Oxfordshire, UK) and Aβ_42_ (#KMB3441: Life Technologies, Carlsbad, CA). Frozen tissue samples were homogenized via sonication (Branson Digital Sonifier; 10% amplitude for two 2-s pulses) in buffer (for pNF-H: 4 M Urea, 1 mM EDTA, 1 mM EGTA, 0.2 mM PMSF, with 1X Halt protease and phosphatase inhibitors; for Aβ_42_: 5 M Guanidine-HCl in Dubellco's PBS, 0.2 mM PMSF, and 1X Halt protease inhibitor cocktail). Homogenate volume was approximately 100 μl buffer per mg of tissue. Homogenates were centrifuged at 15,200 × *g* and 4°C for 5 or 20 min, for pNF-H and Aβ_42_ samples, respectively. Supernatants were decanted into fresh Eppendorf tubes. Samples were diluted 1:50 with the EnCor protein block for pNF-H assays or 1:20 in PBS for Aβ_42_ assays and the assays were carried out according to the manufacturers' protocols. Protein standards were provided in each ELISA kit. Briefly, antibody-coated microplates were incubated with 100 μl of sample, standard, or background (assay buffer/diluent buffer) at room temperature (RT) for 2 h. Plates were washed three times with wash buffers provided in kits, then 100 μl of detection antibody was added to all wells and incubated for 1 h at RT. Plates were washed 3 times and 100 μl of HRP-conjugated secondary antibody was added to all wells and incubated for 1 h. After three washes, 100 μl of 3,3′,5,5′-tetramethylbenzidine (TMB) substrate was added to every well and incubated at RT for 25–30 min. Stop solution (2N H_2_SO_4_) was added and plates were read at 450 nm on a SpectraMax 340 PC plate reader (Molecular Devices, Sunnyvale, CA) using SoftMax Pro 5.2 analytical software. Mean absorbance values were recorded and calculated concentrations were based on the standard curve.

### Tau multiplex

Phosphorylated tau (ptau-231; phosphorylated at the threonine 231 site) and total tau proteins were measured using magnetic bead-based neurodegeneration multiplex plates for the Luminex platform (HND1Mag-39K; EMD Millipore, Billerica MA). Fresh tissue samples were homogenized via sonication in T-Per buffer supplemented with Halt protease and phosphatase inhibitors and centrifuged at 1000 × *g* for 10 min. Supernatants were collected for further analyses; pellets were re-suspended with 50–100 μl of additional T-Per buffer and were centrifuged a final time (1000 × *g*, 10 min, 4°C) with supernatants added to final sample volume. Multiplex analyses were conducted according to manufacturer's instructions. In brief, a solution containing antibody-coupled beads for total tau and ptau-231 were pipetted into a 96-well microplate (25 μl/well). Twenty-five microliters of assay diluent was then added, followed by 25 μl of sample or appropriate standard. Plates were light-protected and incubated for 16–18 h on an orbital shaker (500–600 rpm) at 4°C. Following incubation, plates were washed three times with manufacturer-provided detergent solution using a handheld magnet to keep beads in place. Twenty-five microliters of HRP-conjugated detection antibody was added to all wells and incubated at RT for 1 h (shaking at 500–600 rpm). Plates were washed three times as described and 25 μl of Streptavidin-RPE was added to wells. After a final wash, 100 μl of Magpix drive fluid was added to wells, plates were vigorously shaken for 3 min, and then plates were read on a Magpix Luminex 200. Xponent software was used to generate a standard curve for each analyte from which concentrations of unknown samples were calculated.

### Automated capillary-based western blotting for SBDP and β-tubulin

Analyses for SBDP and β-tubulin were performed using Wes, an automated capillary-based Western blotting platform (ProteinSimple, Santa Clara, CA). Brain samples used for Wes were selected from DBA/2J mice demonstrating either intact CTB transport (no visible deficit in the SC) or 0% transport (no detectable CTB label in the SC). T-Per buffer containing Halt protease and phosphatase inhibitors was added to samples (approximately 10 μl per 1 μg of tissue), tissue was then homogenized via sonication (20% amplitude for two, 2-s pulses), and brain homogenates were centrifuged at 14,000 rpm for 10 min at 4°C. Wes procedures were performed according to manufacturer protocol. Briefly, brain or ON homogenate was diluted to 0.1 μg/μl in 0.1X sample buffer and a fluorescent master mix (1 μl per 5 μl total volume) was added; the sample solutions and ladder were then placed in a thermocycler and heated to 95°C for 5 min and subsequently cooled to 25°C. The samples, blocking reagent, wash buffer, primary antibodies, HRP-conjugated secondary antibodies, chemiluminescent substrate, and separation/stacking matrices were dispensed into a pre-filled microplate (ProteinSimple, PS-PP03). After plate loading, immunodetection was fully automated using default instrument settings for size-based assays. Primary antibodies used were SBDP (mouse, 1:20; Santa Cruz, Dallas, TX) and β-tubulin (rabbit, 1:200: Covance/BioLegend, San Diego, CA). GAPDH (1:100: Sigma-Aldrich, St. Louis, MO) was used as a loading control. All antibodies were diluted with antibody diluent (ProteinSimple, 042-195). Resulting chemiluminescent signals were quantitated and analyzed with Compass software (ProteinSimple v2.5). Protein densitometry was calculated by dividing the area under the curve of each protein of interest by area under the curve of the loading control (GAPDH).

### Bicinchoninic acid assay for total protein

Total protein content was assessed in tissue samples using the Pierce Bicinchoninic Acid (BCA) assay kit (Thermo Fisher; #23227) and individual protein levels were normalized to total protein content within tissue samples. Samples for pNF-H were diluted to a urea concentration compatible with the assay (<3 mM).

### Calculating percent intact transport

Whole-mount SC were imaged with the Zeiss AxioZoom microscope to capture the full extent of the dorsal surface of the SC. Captured images were imported into ImagePro Premier (Media Cybernetics; Rockville, MD) software where areas containing CTB signal were defined within the total SC area. To quantify percent intact transport of each SC, the area of intact CTB transport was divided by the total collicular area (Wilson et al., 2015).

### Immunofluorescence

Sections from fixed retina, brain, and longitudinal ON were blocked with 5% normal donkey serum and 0.1% Triton-X 100 in PBS for 2 h and then incubated for 48 h at 4°C with the primary antibody cocktail (diluted in 3% serum, 0.1% Triton in PBS). Primary antibodies used consisted of the following: mouse anti-SMI-310 (superphosphorylated NF-H 1:1000; AbCam, Cambridge, MA), rabbit anti-tau p231 (1:400; Life Technologies), or rabbit anti-tubulin β-III (1:1000; BioLegend), and goat anti-Brn3a (1:400; Santa Cruz) for identification of retinal ganglion cell bodies. Staining for “superphosphorylated” NF is distinguished from the more typical “hyperphosphorylated” phosphoisoforms seen with other pNF antibodies (e.g., SMI-31, SMI-34, RT97) in that the former represents the highest level of phosphorylation of all the antibody-detected NF phosphoisoforms, and is typically only found in a subset of axons in normal tissue. Assessment of superphosphorylated NF allows us to identify pathological changes in the axon that might be masked by the relatively high expression of other NF phosphoisoforms in normal tissue. Following primary antibody incubation, tissue was washed in PBS and Alexa Fluor secondary antibodies (Jackson Immunoresearch Laboratories, West Grove, PA) against mouse, rabbit, and/or goat (diluted 1:250 in PBS containing 0.1% Triton X-100 and 1% normal donkey serum) were then added and sections were incubated for 2 h at room temperature. Tissue was then washed with PBS, mounted onto slides and cover-slipped with Fluoromount-G (Southern Biotech, Birmingham, AL) prior to visualization.

### Semi-thin optic nerve embedding, sectioning, and immunofluorescence

Perfusion-fixed ONs were immediately placed in 1% glutaraldehyde for 3 h and were then washed in buffer (3.5% sucrose/50 mM glycine in 0.01 M PBS) for 10 min. Tissue was dehydrated using cold 70% ethanol followed by 95% ethanol, and 100% ethanol each for 5 min. Tissue was then placed in a (1:1) mixture of LR white resin (#62662; Sigma-Aldrich) and 100% ethanol for 5 min. After removal from mixture on day 2, tissue was placed in fresh LR white twice, for 5 min each, prior to overnight incubation in LR white, at 4°C. On day 3, tissue was added to gelatin capsules (#7114, Electron Microscopy Sciences, Hatfield, PA) filled with LR white, covered, and placed in an oven at 50°C for overnight infiltration.

Using a Leica UC6 ultramicrotome (Buffalo Grove, IL) equipped with a Diatome diamond knife (Ultra 45, 3.0 mm), 1 μm cross-sections were collected and mounted onto subbed glass slides. Tissue was blocked with 5% normal donkey serum and 0.1% Triton-X 100 in PBS for 2 h. Tissue incubated for 48 h at room temperature in primary antibody solution (3% serum, 0.1% Triton in PBS) consisting of anti-β-tubulin III (1:50; BioLegend) and anti-SMI310 (1:400; AbCam). Following primary antibody incubation, tissue was washed in PBS (10 min, x3) before secondary antibody incubation using Alexa Fluor secondary antibodies (Jackson Immunoresearch Laboratories) against mouse, rabbit, and/or goat (diluted 1:200 in PBS containing 0.1% Triton X-100 and 1% normal donkey serum) overnight at room temperature. Tissue was then washed with PBS before slides were cover slipped using Fluoromount-G mounting media.

### Immunofluorescence imagining

Immunofluorescence imaging of retina, SC, and cross-section ON was completed using a Zeiss Axio Imager M2 epifluorescent microscope outfitted with an Apotome.2 for obtaining optically sectioned images, digital high resolution camera (AxioCam MRm Rev.3), and motorized Z and X-Y stage (Zeiss, Jena, Germany). Longitudinal ON were imaged using the Zeiss AxioZoom V16 epifluorescent microscope.

### Tau-dynactin co-immunoprecipitation

To test the effect of tau hyperphosphorylation on its affinity for the retrograde motor component, dynactin, we performed co-immunoprecipitation comparing native tau (that exhibits a low level of phosphorylation) and tau hyperphosphorylated by pretreatment with glycogen synthase kinase 3β (GSK-3β; a kinase that has tau as one of its major substrates). In a 1.5 ml Eppendorf tube, 30 μl of Dynabeads-G (ThermoFisher, #10003D) was bound with 10 μl of antibody against dynactin-2 (AbCam, #EPR5095), which was then allowed to bind 5 μg recombinant dynactin-2 (0.5 μg/μl; OriGene Technologies Inc., Rockville, MD; #TP314771). The tubes were rotated for 20 min at RT to ensure thorough mixing. Five micrograms of human tau-412 isoform (1 μg/μl; rPeptide, LLC, Bogart GA), was treated with either GSK-3β, lambda phosphatase, or PBS and incubated at RT for 30 min. The tau solutions were then mixed with dynactin-bound Dynabeads and allowed to rotate for 20 min. Fifty microliters of Laemmli buffer was added to each reaction and tubes were heated at 70°C for 10 min. Forty microliters of each solution was used to perform Western blots which were run on 4–15% Mini-Protean TGX precast gels (Bio-Rad, Hercules, CA; #4561083SEDU) and Immun-Blot PVDF membranes (Bio-Rad; #1620177). Membranes were probed with an antibody against pan-tau (anti-Tau-5; AbCam #ab80579) to determine relative amounts of total tau pulled out in each reaction context. Blots were stripped (Restore™ Western Blot Stripping Buffer; ThermoFisher; #21059) and probed with a phospho-specific antibody (phospho-(Ser/Thr) Phe Antibody; Cell Signaling, Anvers, MA; #9631) to confirm differences in phosphorylation state. Control blots were run using either Dynabeads-only (30 μl), pure dynactin, or native tau protein (1 μg).

### Statistical analyses

IBM SPSS Statistics 22 (IBM Corp: Armonk, NY, USA) was used for all statistical analyses. To justify pooling D2G results into a unitary control group, two-tailed, independent samples student *t*-tests were performed to confirm similarity of results between young (3–5 months.) and old (12–15 months.) D2G control animals. Assessments regarding whether pooling was applicable were made separately for each protein (pNF-H, ptau, total tau, and Aβ_42_) and tissue type (retina, ON, SC, and cerebellum). Independent samples *t*-tests were performed to determine any initial sex differences in protein quantification measurements within each strain/age group of mice prior to assessment of our overall hypotheses. To test our overall hypotheses, we conducted one-way analyses of variance (ANOVA) using protein concentration (pNF-H, ptau, total tau, and Aβ_42_) per anatomical region (retina, ON, SC and cerebellum control) as the dependent variable and strain/age group as the independent variable. Bonferroni-corrected *post hoc* comparisons were performed to elucidate specific group differences. For other analyses, we used transport outcome as our independent variable to determine whether proteins levels in each region differed in transport-intact (CTB-positive) vs. transport-deficient (CTB-negative) projections. Since the distribution of projections with intact transport was variable in the D8-10 DBA/2J group (53% intact) and skewed substantially toward loss in the D12-15 group (15% intact), we pooled data across these two age groups to have sufficient cases to support each level of our dichotomous transport-intact/deficient variable for analysis. In addition to this dichotomous transport variable, we also used a continuous variable of “percent intact transport” for our correlational analyses.

## Results

### D2G mice show no differences in protein concentrations based on age

To assess whether age-related differences in cytoskeletal components occur independent of strain, we compared pNF-H protein levels in retina, ON, SC, and cerebellum (control structure) between young (3–5 months.) and old (12–15 months.) D2G using independent samples *t*-tests. No significant differences in pNF-H levels were shown between young and old D2G for any structure (refer to Table 2). These results were recapitulated in analyses of Aβ and tau proteins as well. Therefore, young and old D2G were pooled into a single control group for all subsequent analyses. No significant sex differences were observed within either D2G age group.

**Table 2:** **Young and aged D2G comparisons**.

**Protein**	**Structure**	***t***	**Mean Difference**	***p*-value**
**pNF-H**	*Retina*	0.316	0.001547	0.757
	*ON*	2.28	0.063000	0.063
	*SC*	0.558	0.011460	0.578
	*Cerebellum*	−0.073	0.001700	0.949
**A**β**42**	*Retina*	0.688	0.749250	0.501
	*ON*	2.016	2.590410	0.075
	*SC*	0.424	0.753093	0.681
	*Cerebellum*	0.237	1.493809	0.834
**Ptau-231**	*Retina*	−0.64	0.002750	0.568
	*ON*	0.873	0.024179	0.447
	*SC*	−0.826	0.001414	0.561
**Total tau**	*Retina*	−0.534	0.000016	0.631
	*ON*	1.083	0.000459	0.358
	*SC*	−0.983	0.000000	0.506

### pNF-H levels in DBA/2J mice are elevated in an age and transport dependent manner

#### DBA/2J mice show early pNF-H elevations in SC and late increases in the retina, with transitional changes occurring in ON

**Figure 1:**
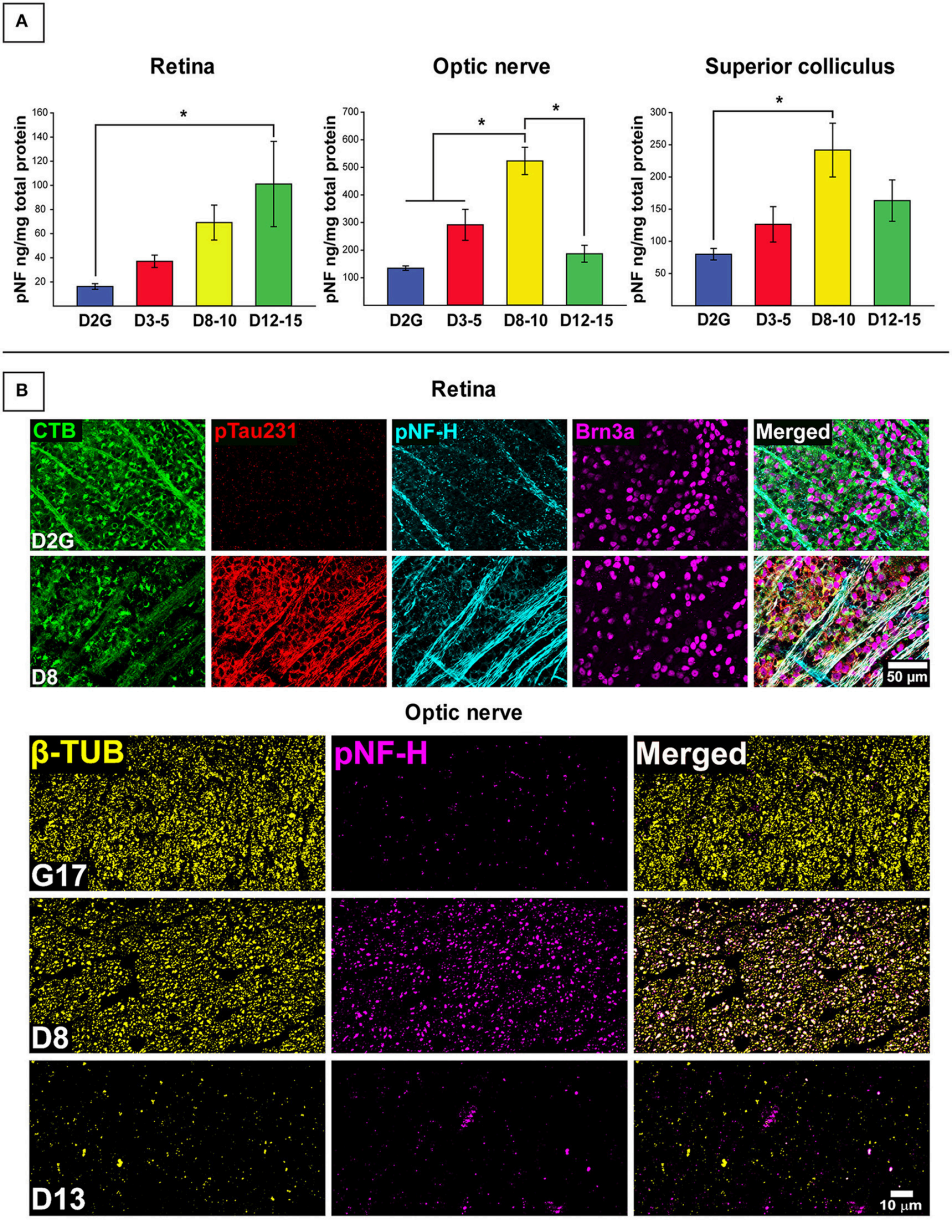
**Early elevations in pNF-H observed in distal RGC projection with effects of age and transport outcome. (A)** Quantification of pNF-H by ELISA in retina, optic nerve (ON), and superior colliculus (SC). DBA/2J retinal pNF-H levels show trending increase with age peaking at 12–15 months at which point, is significantly elevated compared to the D2G controls. In the ON, pNF-H levels were highest at 8–10 months. in the DBA/2J mice but returned to levels similar to the D2G controls at 12–15 months. In the SC, pNF-H levels remained significantly elevated in the D8-10 group compared to controls. **(B)** (Top) Immunofluorescence of cytoskeletal markers, SMI-310 (specific for superphosphorylated, pNF-H) and the RGC marker Brn3a in the retina of an 8-mo. DBA/2J (D8) and D2G control. Micrographs show visibly increased pNF-H and somatic/axonal ptau-231 staining in the D8 retina. The D2G pNF-H labeling is typical of mature, non-pathological axons within the retina. (Bottom) Semithin (1 μm) cross-sections taken from 8-months. DBA/2J (D8), 13-months. DBA/2J (D13), and 17-months. D2G (G17) control ON immuno-stained for β-tubulin (β-TUB) and pNF-H. A significant increase in pNF-H is observed in the D8 ON compared to G17 control. In the D13, pNF-H in the ON is nearly absent corresponding to the prominent loss of axonal structure indicative by the reduction in β-TUB. Error bars depict SEM. Asterisk indicates significant statistical difference (*p* <0.05).

Figure 1 depicts results for pNF-H analyses. As shown in Figure 1A, pNF-H protein concentration differed between strains in the retina [*F*_(3, 55)_ = 3.116, *p* = 0.034], ON [*F*_(3, 52)_ = 17.206, *p* <0.001], and SC [*F*_(3, 50)_ = 3.653, *p* = 0.019] with no differences in cerebellar control tissue (Table 2). An approximate 5-fold increase in collicular pNF-H was seen in the 8–10 month DBA/2J group (*p* = 0.020) compared to controls. Retinal pNF-H levels did not significantly differ between groups until DBA/2J mice were 12–15 months of age, whereby pNF-H levels were increased by nearly 5-fold in 12–15 month DBA/2Js compared to controls (*p* = 0.033). pNF-H results in the ON showed a transitional pattern of results, with D8-10 mice displaying significantly higher pNF-H levels—more than double—in comparison to D2G controls and D3-5 (*p* = 0.003) and D12-15 (*p* <0.01) mice. Further, we saw elevations of pNF-H in the most superficial SC between groups (Figure 1A). Following the pattern of ON NF levels, we see an increase of pNF in D8-10 followed with a reduction in the oldest age group. Figure 1B shows immunofluorescent labeling of superphosphorylated NF-H (SMI-310) in retina and ON. Retinas show low levels of pNF and ptau in the control retina with the D8 retina showing greatly increased levels of pNF and ptau, with both proteins being aberrantly localized in the RGC somata (labeled with Brn3a), a sign of pathology. Immunofluorescence staining in our semithin cross-sections of ON is also consistent with results from the protein quantification. We found low levels of pNF-H in aged control mice with very robust staining evident in a D9 ON. This staining is greatly reduced in the D13 ON, likely due to axon loss, as evidenced by the massive reduction in β-tubulin seen in the same section. No sex differences were observed within retina, ON, or SC of any DBA/2J age group.

#### pNF-H levels in retina and SC vary based on transport outcome in DBA/2J mice

**Figure 2:**
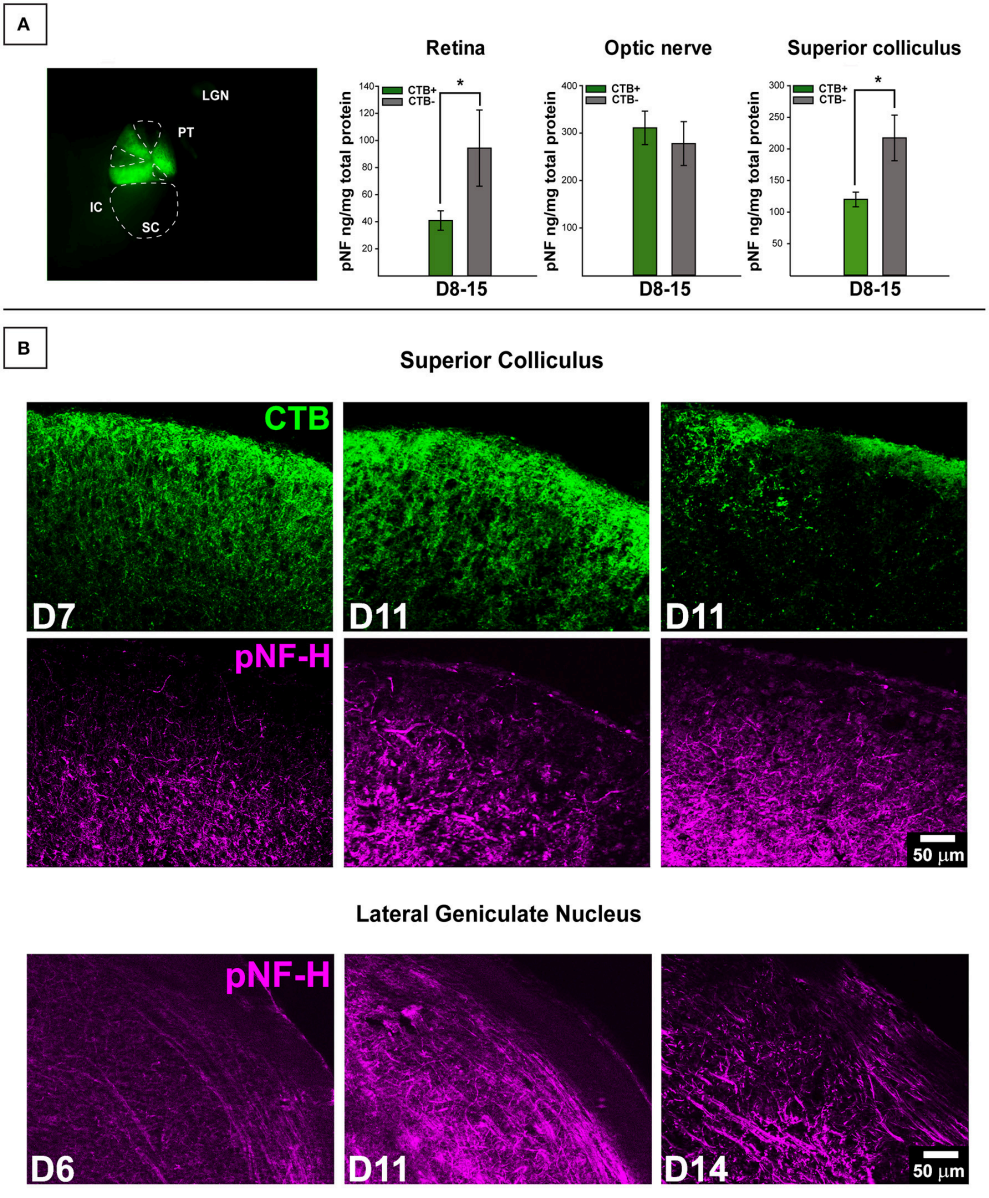
**Transport-dependent elevations in pNF-H observed in retina and SC of DBA/2J mice. (A)** (Left) Photomicrograph from the dorsal surface of a whole-mount pathological SC in which the cortex has been removed and cholera toxin-B (CTB) labeling shows sectorial loss in the left (top) collicular lobe and complete loss in the right (bottom) lobe. Abbreviations: IC, inferior colliculus; PT, pre-tectum; LGN, lateral geniculate nucleus; SC, superior colliculus. The dotted lines demarcate areas absent of CTB indicating axonal transport deficit (CTB-). (Right) ELISA quantification of pNF-H in retina, optic nerve (ON), and SC. The transport status of retina and ON were determined by whether or not the SC was CTB+ (>90% CTB-label for partially transporting projections) or CTB- (<10% CTB-label for partially transporting projections). Levels of pNF-H were significantly elevated nearly 2-fold in both retina and SC of CTB- projections compared to CTB+ projections. Asterisks indicate significant statistical differences (*p* <0.05). Error bars depict SEM. **(B)** Top panel shows immunofluorescence of the SC, comparing 7-months. (D7), and 11-months. (D11) DBA/2J with and without CTB transport. Visibly increased pNF-H is shown in CTB- D11 SC. Bottom panel illustrates an age-dependent increase in pNF-H staining in the LGN (the visual thalamus) of 6, 11, and 14-month DBA/2J mice.

Collicular tissue was microdissected to separate CTB+ from CTB- areas, each area was run separately. For retina, total CTB+ area of corresponding collicular lobe was used for classification. We contrasted pNF-H levels in retinal projections taken from mice with deficient (0–20%) anterograde transport (CTB-) in the SC with projections taken from SC displaying full tracer coverage (CTB+) and these data are presented in Figure 2. Only DBA/2J mice in the D8-10 and D12-15 groups displayed transport deficits (as anticipated based on the established trajectory of transport loss in this model) with only 15% of D12-15 colliculi exhibiting any detectable CTB label whatsoever. To maximize detection of differences in pNF-H levels as a function of transport outcome, we pooled data across the two pathologically-aged DBA/2J groups (D8-10 and D12-15). Significant elevations of pNF-H were indicated in both SC [*F*_(1, 53)_ = 8.288, *p* = 0.006] and retina [*F*_(1, 59)_ = 5.816, *p* = 0.019] of transport deficient (CTB-) projections compared to intact (CTB+) projections (Figure 1A). There were no differences in ON or cerebellum levels (not shown) of pNF-H as a function of transport outcome. For both SC and retina, the percent of intact transport was negatively correlated with pNF-H levels indicating that pNF-H levels increased as a function of transport deficit magnitude in these structures (SC: Pearson *r*^2^ = −0.358; retina: Pearson *r*^2^ = −0.317, *p* <0.05 in both cases). pNF-H levels in the ON did not show this same relationship to transport deficit magnitude. There were no observed differences in transport outcome to the SC based on mouse sex.

### Phosphorylated tau shows age-dependent changes in retina and ON

#### Age-dependent elevations in ptau in DBA/2J mouse SC and retina

**Figure 3:**
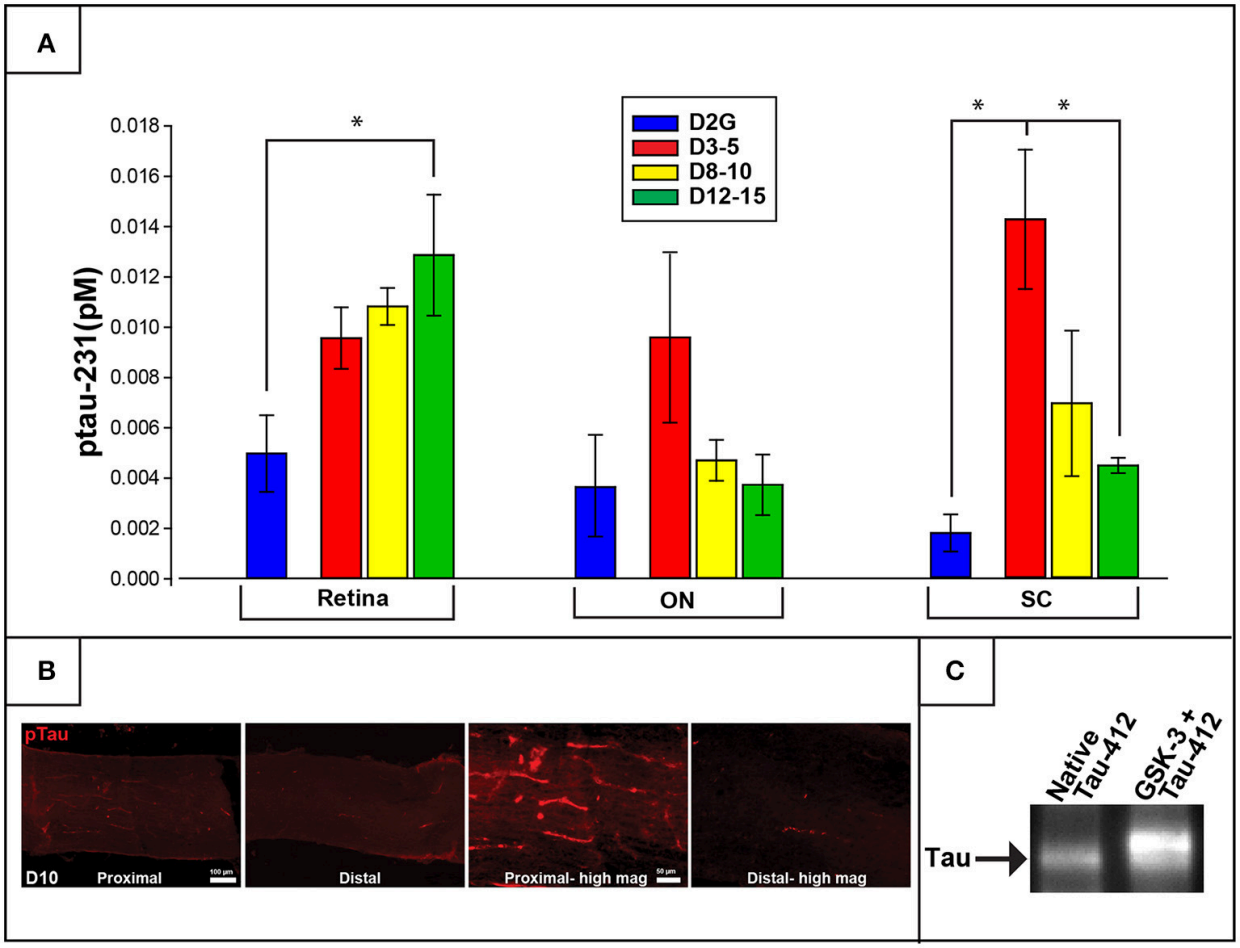
**Phosphorylated tau (ptau-231) is elevated early in the distal projection and is observed to translocate to the retina in pathologically-aged animals**. **(A)** Multiplex quantification of ptau-231 in DBA/2J mice shows an age-dependent decrease of ptau in SC with an age-dependent increase in retinal ptau. In the SC, ptau-231 is significantly higher in the D3-5 group compared to D12-15 and D2G control group levels. In retina, however, D12-15 ptau-231 levels are significantly higher than D2G controls. Ptau levels in ON samples did not differ between groups. **(B)** Immunofluorescence for ptau-231 in 10-months. DBA/2J ON shows evidence of preferential accumulation of ptau-231 in the proximal portion compared to the distal portion of the nerve. **(C)** An example of co-immunoprecipitation data comparing native and hyperphosphorylated tau via reaction with GSK-3 suggests an increased affinity of phosphorylated tau for the retrograde motor component, dynactin-2. This is qualitatively illustrated by the increased optical density of Tau-5 (a marker for pan-tau) in the second lane representing hyperphosphorylated tau (GSK-3+Tau-412), compared to the first lane which contained only native tau. The tau band shown here corresponds to a molecular weight of approximately 43.5 kDa corresponding to the GSK-3 phosphorylation site, pS396/404. Asterisk indicates that groups are significantly different from bracketed comparison (*p* <0.05). Error bars depict SEM.

Concentrations of ptau-231 differed as a function of DBA/2J age group in retina [*F*_(3, 22)_ = 3.850, *p* = 0.026] and SC [*F*_(3, 16)_ = 7.209, *p* = 0.004] but not in ON or control cerebellar tissue. Specifically, collicular ptau-231 was elevated by more than 4-fold in the youngest DBA/2J group (D3-5) compared to D2G controls (*p* = 0.007) and D12-15 mice (*p* = 0.011) (Figure 3). In contrast, retinal levels of ptau-231 were significantly increased about 3-fold in the D12-15 group compared to D2G controls (ptau-231; *p* = .022) and displayed a stepwise pattern of age-related increase among DBA/2J mice (Figure 3A). As shown in Figure 2B, there was a notable elevation in ptau-231 immunofluorescent labeling in the somata and axons of an 8-month old DBA/2J retina compared to retina from an 11-month old D2G control, despite no differences in the distribution of the RGC marker Brn3a between retina of these mice. Although differences in ptau-231 protein levels between groups were not detected in of ON homogenates, immunofluorescent ptau labeling in fixed longitudinal ON sections of 14-month old DBA/2J showed an intriguing distal-to-proximal distribution. Ptau-231 label was obvious in myelinated proximal ON but was sparse in the distal portion of the nerve (Figure 3C), and this pattern was observed in three cases of D14 ON and not in any other age groups. Relationships between transport deficits and ptau-231 protein levels could not be determined due to high variability in tau protein concentrations and small sample sizes of transport-intact mice in the oldest DBA/2J age groups. No sex differences were observed within retina or SC of any DBA/2J age group.

#### Tau-dynactin co-immunoprecipitation

To further explore the age-related distal-to-proximal distribution of ptau in the DBA/2J retinal projection, we conducted a co-immunoprecipitation assay for differences in binding affinity of different tau phosphoisoforms with dynactin-2, the link between the retrograde molecular motor dynein and cytoskeletal elements. Results indicated that more tau protein was pulled down by the dynactin protein after GSK-3β-mediated phosphorylation compared to native conditions or phosphatase pre-treatment, as observed by Western blot (Figure 3C). Dynabead-only, tau, or dynactin alone conditions showed little if any non-specific binding. Blots were stripped and probed with a phospho-specific antibody to confirm differences in phosphorylation state (not shown).

### Elevations in SBDP observed early in ON and SC of DBA/2J mice

**Figure 4:**
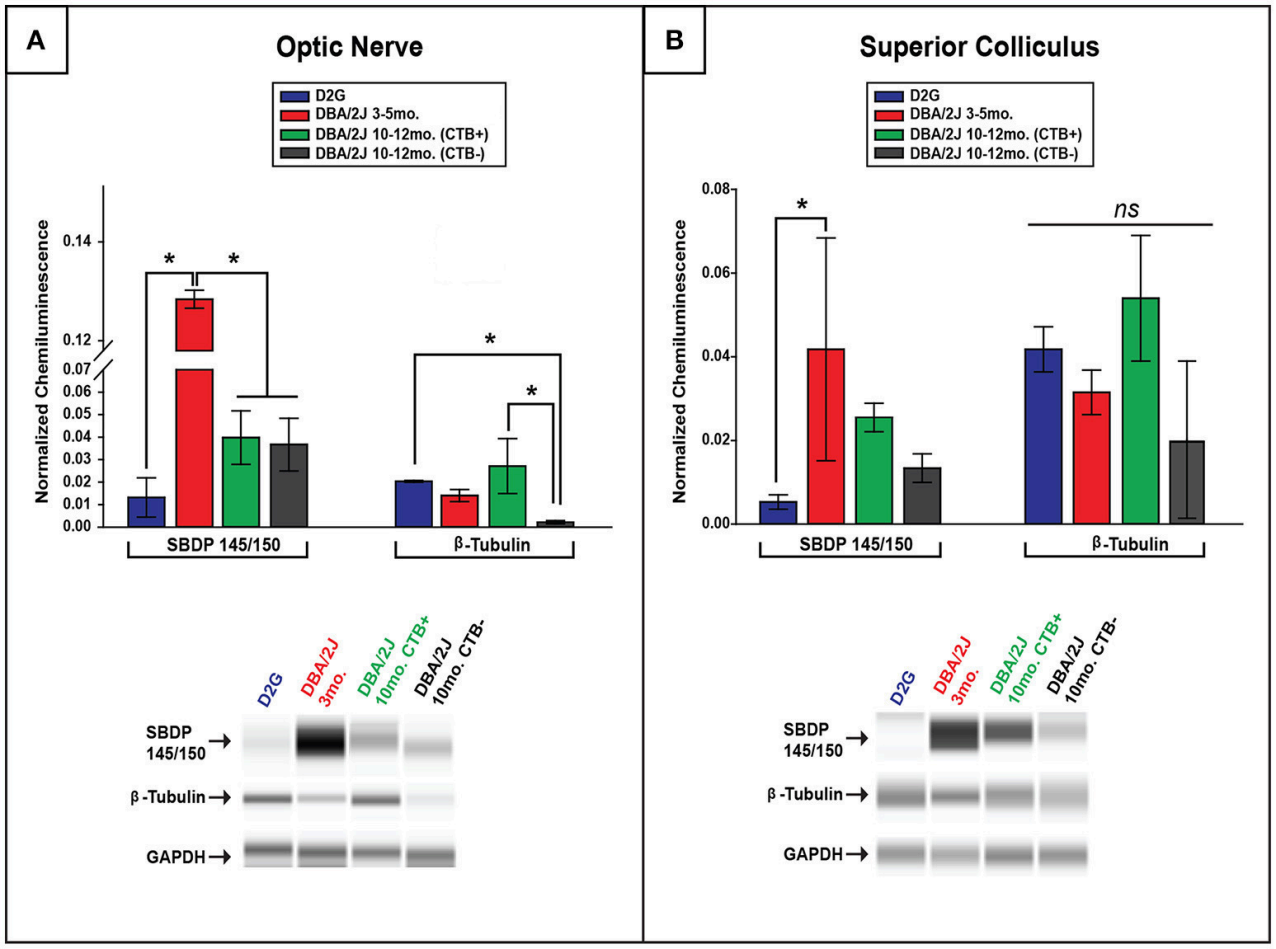
**Spectrin breakdown product (SBDP) 145/150 is elevated in young DBA/2J ON and SC while β-tubulin is decreased in a transport-dependent manner in pathologically-aged ON**. **(A)** Chemiluminescence-based quantification using ProteinSimple Wes technology illustrates significantly higher levels of SBDP(145/150) within ON of D3-5 mice compared to all other groups, with no differences seen in pathologically-aged (10–12 months.) DBA/2J mice regardless of transport outcome. Bar graph (top) shows SBDP145/150 and β-tubulin normalized to GAPDH (a housekeeping gene marker), while corresponding examples of automated western output are shown below for ON. Importantly, a significant decrease in β-tubulin was observed in pathologically-aged ON associated with zero anterograde transport to the corresponding SC compared to controls and D10-12 mice with intact transport. **(B)** A significant elevation in SBDP chemiluminescence was observed in D3-5 SC compared to D2G controls **(B)**, with no differences in collicular β-tubulin elucidated. Bar graph (top) shows SBDP145/150 and β-tubulin normalized to GAPDH, while corresponding examples of automated western output are shown below for SC. (Refer back to Figure 1 to see β-tubulin loss using immunofluorescence.) Error bars depict SEM. Asterisk indicates that groups are significantly different from bracketed comparison (*p* <0.05)

Comparisons of spectrin breakdown product (SBDP) protein levels were only performed between groups of D2G, D3-5, and D10-12 DBA/2J mice with this oldest group exhibiting a bimodal distribution in terms of transport outcome (CTB+/CTB-). SBDP levels varied significantly between these groups in the ON [*F*_(3, 13)_ = 32.36, *p* <0.01] and SC [*F*_(3, 13)_ = 4.718, *p* = 0.027]. Interestingly, D3-5 DBA/2J mice demonstrated significantly elevated SBDP in ON compared to all other groups (*p* <0.01 for all comparisons; Figure 4A), a difference of more than 10-fold. These young DBA/2J mice also expressed collicular levels of SBDP that were more than double the levels detected in D2G controls (*p* <0.05; Figure 4B). As expected based on this result, SBDP protein levels did not differ as a function of transport outcome in the D10-12 group exhibiting either fully intact or completely absent transport to individual SC. No sex differences were observed within ON or SC of any DBA/2J age group.

### Decreased β-tubulin in pathologically-aged DBA/2J mice with disrupted transport

To determine whether lower levels of SBDP observed in ON and SC of 10–12 month old DBA/2J mice were potentially due to an overall loss or disorganization of structure in aged projections, we quantified β-tubulin (a major structural component of microtubules) in these structures. In 10–12 months old DBA/2J mice, β-tubulin was significantly decreased within the ON of transport deficient (CTB-) projections relative to transport intact (CTB+) projections [*F*_(1, 11)_ = 9.11, *p* = 0.01], and transport-deficient D10-12 projections also had lower levels of β-tubulin levels compared to D2G projections [*F*_(3, 11)_ = 5.053, *p* = 0.030] (Figure 4A). Immunofluorescent staining in the ON also reflected decreased β-tubulin and CTB label in 11-month old DBA/2J tissue compared to 6-month old DBA/2J ON (Figure 1B). No significant differences in collicular β-tubulin levels were detected between any groups (Figure 4B); however, β-tubulin levels in this region showed trends similar to those observed in the ON. No sex differences were observed within ON or SC of any DBA/2J age group.

### Elevations in Aβ_42_ levels in DBA/2J

#### Retinal Aβ_42_ levels are elevated in all DBA/2J mice regardless of age, but show age-dependent increases at the level of the SC

**Figure 5:**
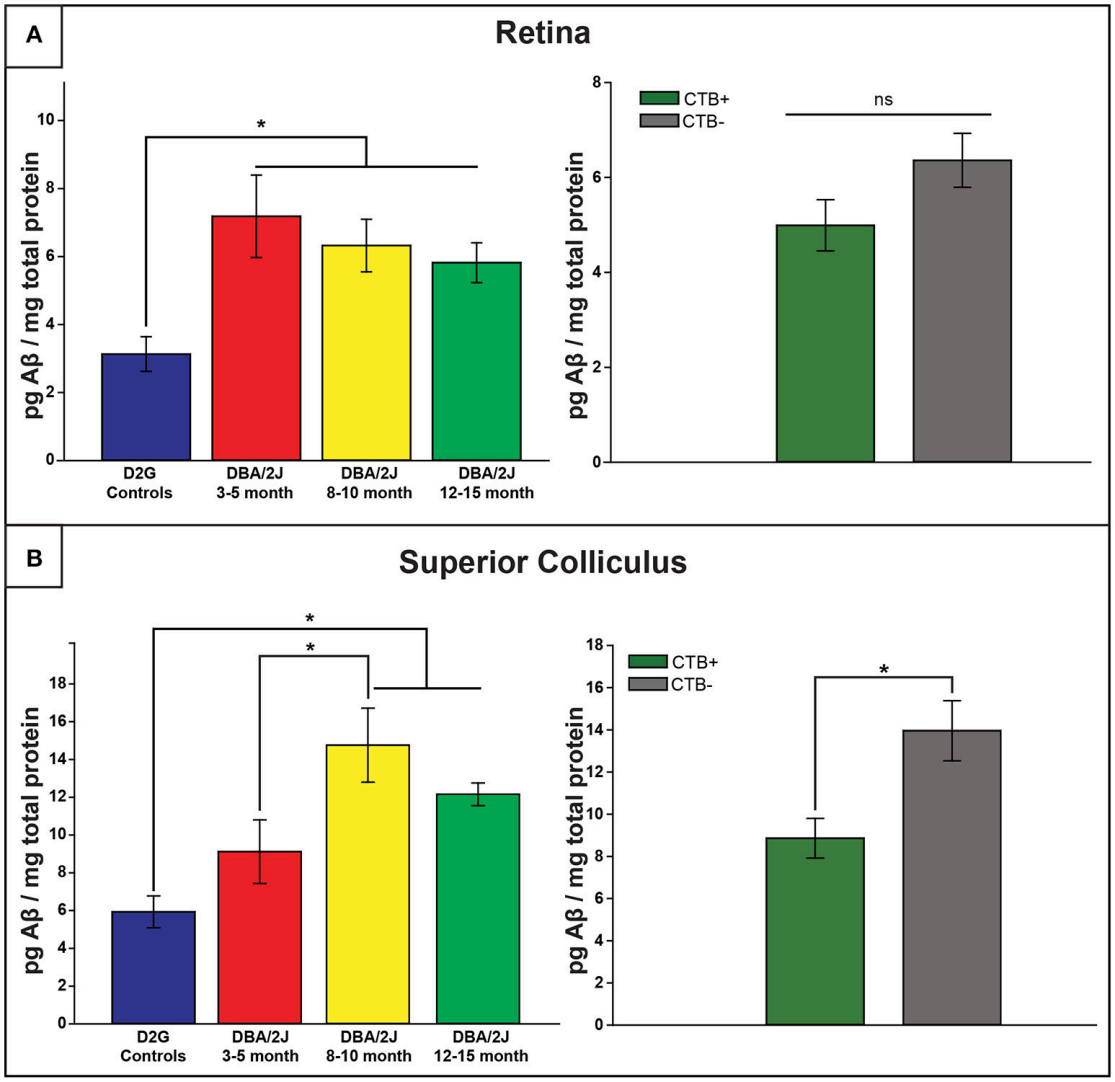
**Amyloid-β_**42**_ elevations provide an early retinal marker of pathology and are later observed in the distal projection**. **(A)** Retinal Aβ_42_ was elevated in all DBA/2J age groups compared to controls (left), with no transport-dependent effects observed (right). **(B)** However, collicular elevations appeared later, with 8–10 and 12–15 month DBA/2J SC having significantly elevated levels compared to controls; a significant difference was also observed between 3–5 and 8–10 month DBA/2Js (left). Levels of Aβ_42_ were significantly elevated in SC lacking intact transport (CTB-) compared to transport-intact (CTB+) SC (right). Error bars indicate SEM. Asterisk indicates that groups are significantly different from bracketed comparison (*p* <0.05).

Retinal and collicular Aβ_42_ levels varied as a function of strain, age, and location [retina: *F*_(3, 61)_ = 5.886, *p* <0.01; SC: *F*_(3, 38)_ = 7.760, *p* <0.01]. Retinal Aβ_42_ was elevated in all age groups of DBA/2J mice, by three to four times, relative to D2G controls (*p* ≤ 0.010 in all analyses; Figure 5A, top). Collicular Aβ_42_ was significantly elevated in D8–10 and D12–15 mice by more than double control levels, but not in the young D3–5 group (*p* <0.05 in both cases; Figure 5B, top). In DBA/2J SC, Aβ_42_ was highest in the D8–10 group, although statistically these Aβ_42_ values only differed from the D3–5 group (*p* = 0.03). Aβ_42_ levels in cerebellar tissue did not differ by age or strain. No sex differences were observed within retina or SC of any DBA/2J age group.

#### Aβ_42_ levels are elevated in DBA/2J SC as a function of transport loss

As illustrated in Figure 5B (bottom), Aβ_42_levels were increased in SC of transport-deficient (CTB-) projections relative to transport-intact (CTB+) projections of DBA/2J mice (collapsed across D8–10 and D12–15 groups) [*F*_(1, 41)_ = 8.044, *p* = 0.007]. No transport-related differences were found in retina (Figure 5A, bottom) or ON samples (not shown).

## Discussion

**Figure 6:**
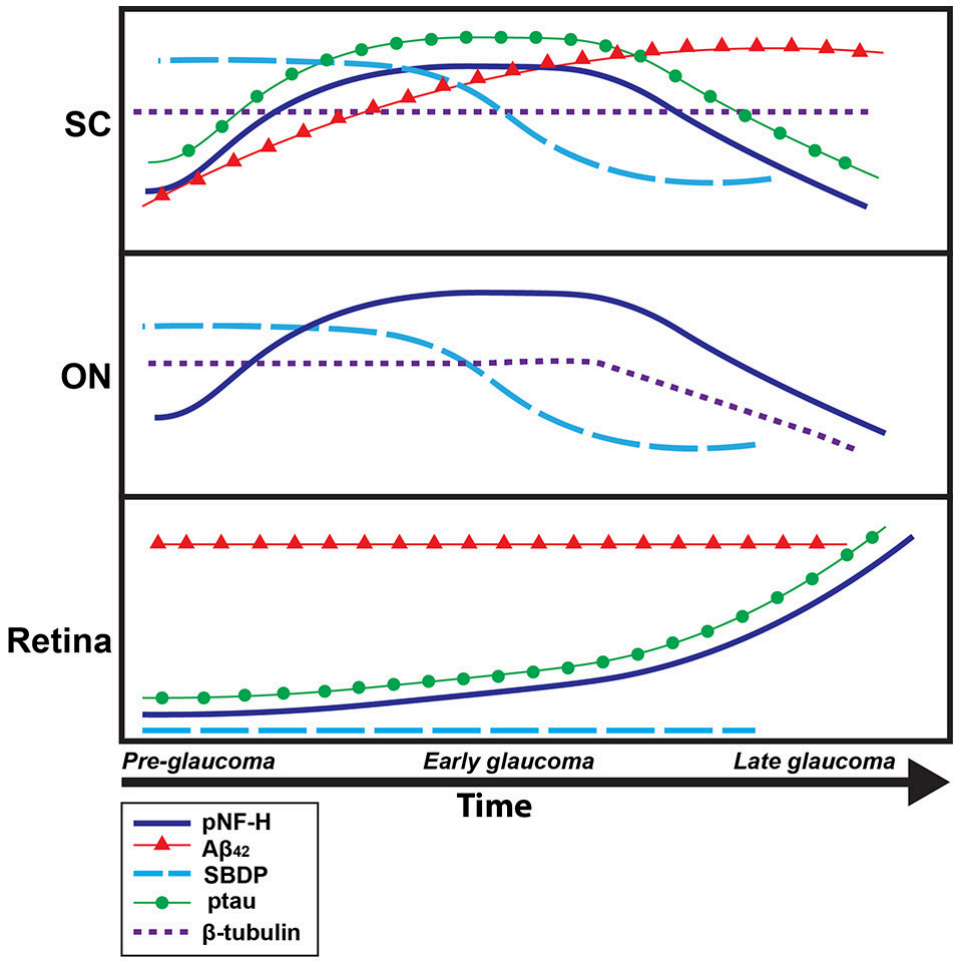
**Timeline summary of protein pathology within the DBA/2J retinal projection**. A summary schematic of changes in protein levels over disease progression (pre-glaucomatous, early, and late glaucomatous ages), in the DBA/2J mouse model, for each tissue type analyzed (retina, ON, SC) is shown here. The x-axis corresponds to time while the y-axis corresponds to general protein levels. Each colored line corresponds to a specific protein: dark blue corresponds to pNF-H, red corresponds to Aβ_42_, light blue corresponds to SBDP, green corresponds to ptau-231, dotted purple corresponds to β-tubulin. Increased/decreased slope of each line indicates increased/decreased concentrations of each specific protein over time.

Pathological phosphorylation and cleavage of cytoskeletal elements are commonly implicated in chronic neurodegenerative conditions, including glaucoma (Lee et al., 2000; Colucci-D'Amato et al., 2003; Knobloch and Mansuy, 2008; Chidlow et al., 2011). Specifically, roles for pNF-H, ptau, spectrin, β-tubulin, and the pathological protein Aβ_42_ in the development, progression, and even classification and diagnosis of degenerative conditions have been prominent in both the clinical and basic research literature. However, the present experiments are the first to quantify this specific subset of proteins throughout the entire retinal projection of a glaucomatous mouse model; likewise, this study is the first to associate protein quantification with measures of axonal transport from the retina to its primary projection target in mouse, the SC. Our data (summarized in Figure 6) indicate that post-translational phosphorylation of neurofilament heavy-chain and tau occurs with age in DBA/2J mice. Spectrin breakdown in ON and SC precedes these effects, and early elevation of retinal Aβ_42_ was evident in the youngest, pre-pathological age group of DBA/2J mice studied. Consequently, elevations in these proteins occur prior to anterograde transport loss in this well-characterized glaucoma model. When the temporal context of these molecular change are considered, it is plausible that early spectrin cleavage and Aβ_42_ accumulation may perpetuate the sequence of degenerative events by facilitating aberrant cytoskeletal phosphorylation and thus, provoking disease progression (Dixit et al., 2008).

Microtubules are the substrate along which motor proteins carry cargo within axons. Therefore, disruption of microtubule organization inhibits axonal transport and, depending on the location and severity of the disruption, can trigger various destruction programs ranging from distal axonopathy to apoptosis (Cuchillo-Ibanez et al., 2008; Song et al., 2013; Zempel and Mandelkow, 2015). Relevant to our findings, abnormal microtubule distribution has been observed following laser coagulation-induced ocular hypertension in rats (Huang et al., 2011). Pharmacological disruption of microtubules with nocodazole treatment indicated that microtubule disruption is an early clinical event that occurs prior to decreased retinal nerve fiber thickness (Lim and Danias, 2012). Microtubules require a vast network of stabilizing, support, and adaptor proteins to maintain their functional and structured matrix (Ahlijanian et al., 2000; Roy et al., 2005). Neurofilaments, the most abundant component of the mature axonal cytoskeleton, aid in organization and stabilization of the microtubule matrix and other cytoskeletal elements. Phosphorylation of NF-H controls axon caliber (Petzold et al., 2008; Cooper et al., 2016), and hyper- or super-phosphorylation of neurofilament can slow its transport, leading it to accumulate and form aggregates that ultimately block transport (Nixon, 1994; Elder et al., 1998a,b; Julien and Mushynski, 1998; Jung and Shea, 1999, 2004), leading to eventual axon loss (Ackerley et al., 2003; Petzold et al., 2008; Lépinoux-Chambaud and Eyer, 2013). We showed that phosphorylated NF-H is significantly elevated in the SC of DBA/2J mice early (8–10 months) in the progression of glaucomatous pathology, decreasing only when glaucomatous pathology advances (12–15 months). Consequently, we also observed decreased β-tubulin in the ON (and to lesser extent in the SC) of transport-deficient 10–12 month DBA/2J projections compared to projections with intact transport—a finding that suggests loss of microtubule and cellular structure within the ON and SC as degeneration progresses (Schlamp et al., 2006; Crish et al., 2010; Adalbert and Coleman, 2012). Supporting this, our data showed increased pNF-H in the SC and retina of mice with transport deficits compared to mice with intact transport. Phosphorylation of neurofilaments decreases their affinity for tubulin, causing abnormal bundling and disorganization of the microtubule array (Buée et al., 2000; Jung et al., 2005; Zempel and Mandelkow, 2015). Therefore, elevation of pNF could be a driving force for transport loss.

Hyperphosphorylation of the microtubule-associated protein tau–a pathological hallmark of Alzheimer's disease (Iqbal et al., 2016)– promotes abnormal translocation and accumulation of tau in the soma and aberrant assembly of microtubules, driving axonal transport deficits and other neurodegenerative effects (Majid et al., 2014; Morfini et al., 2016). While tauopathy likely plays a mechanistic role in glaucoma, evidence of tau phosphorylation and translocation in a glaucoma model has only recently been uncovered (Chiasseu et al., 2016). The data from our study show: (1) an age-dependent increase in retinal ptau and (2) a large decrease in collicular ptau of glaucomatous DBA/2J animals. Supporting this, we found distal-to-proximal increases in ptau immunofluorescence in the ON of DBA/2J mice, indicating retrograde translocation of tau in glaucoma (Buée et al., 2000; Dixit et al., 2008). Additionally, we showed increased tau phosphorylation (at the threonine-231 residue) in the SC of our pre-pathological 3–5 months. DBA/2J mice—an early effect that preceded significant ptau accumulations in retina. These data suggest that tau translocation from the axon into the somatodendritic compartments occurs as tau becomes increasingly phosphorylated. While the mechanism of this translocation is unknown, it is possible that phosphorylation not only changes tau's affinity for microtubules (Johnson and Stoothoff, 2004) but also increases its affinity for the retrograde motor complex. Supporting this, we demonstrated that phosphorylation of tau increases its affinity for dynactin, a component of the retrograde motor complex that links the molecular motor dynein with its cargo (recall Figure 3C).

Calpain-mediated cleavage of α-II spectrin, has been implicated in secondary neurodegenerative processes (such as those resulting from traumatic brain injury; Yan and Jeromin, 2012) and serves as another indicator of axonal injury and pathology. Cleaved α-II spectrin has been found in varicosities and end bulb swellings (Uryu et al., 2007) and is shown to be upregulated in the retina of rodent glaucoma models with elevated IOP (Huang et al., 2010). Spectrin organizes the subaxolemmal actin scaffold that, among other functions, confers strength to axons (Xu et al., 2013), enabling it to resist mechanical stresses (Elson, 1988; Zhou et al., 1999; Jung et al., 2005; Xu et al., 2013). While we were unable to detect SBDP in any DBA/2J or control retina (in contrast to previous findings by Huang et al. (2010) who found elevated SBDP in an experimentally-induced ocular hypertension model), we did observe high levels of calpain-mediated spectrin breakdown product in the ON and SC of our pre-pathological, young DBA/2J mice. This result was somewhat unexpected, as elevated IOP, transport deficits, and axon loss are not typically present in DBA/2J at 3–5 months. Hence, pre-degenerative, post-translational modifications and breakdown of cytoskeletal elements in DBA/2J mice may make their RGC axons vulnerable to glaucomatous neurodegeneration once IOP increases at later ages (Inman et al., 2006).

Elevations in Aβ_42_ fragments often accompany elevated SBDP and the pathological phosphorylation of tau and NF-H in neurodegeneration (Johnson et al., 2016). Aβ_42_ protein is also implicated in axon transport deficits and axonopathy (Stokin et al., 2005; Chidlow et al., 2011), as well as in response to IOP elevation (Ito et al., 2012). We observed elevated levels of Aβ_42_ in the retina of our youngest DBA/2J mice, suggesting this increase occurs prior to IOP elevation. Levels of Aβ_42_ remained elevated in the retina throughout the stages of pathological progression in DBA/2J, and increased in the oldest DBA/2J age group SC. While Aβ_42_ is not a cytoskeletal protein, it activates kinases such as GSK-3β which phosphorylate NF-H and tau (Muyllaert et al., 2008; Martin et al., 2011a,b), potentially conferring susceptibility to axon pathology.

The interplay between early, proximal elevations in Aβ and post-translational modification of cytoskeletal spectrin and neurofilament proteins may be partly explained by the calcium hypothesis of neurodegeneration (Chidlow et al., 2011; Crish and Calkins, 2011). Elevated intracellular calcium, partially promoted by Aβ-mediated increases in Ca^2+^ conductance (Kawahara and Kuroda, 2000) modulates subsequent calpain activation. Ca^2+^-mediated calpain activation leads to specific cleavage of structural proteins such as neurofilament and spectrin (Crish and Calkins, 2011). This is supported by our observations of elevated calpain-mediated SBDP and Aβ_42_ in young, pre-glaucomatous DBA/2J mice. As these pre-pathological mice do not exhibit elevated IOP at this early stage, these findings suggest that factors other than IOP may cause individuals to be predisposed to developing glaucoma (e.g., subtle changes in kinase activity, excitotoxic stress, or Aβ_42_ load). Recent findings showing early increases in neuroinflammation and oxidative stress are germane here. Neuroinflammation is the earliest change reported in models of glaucoma, often occurring well before neurodegeneration (Wax and Tezel, 2009; Bosco et al., 2011; Howell et al., 2011; Wilson et al., 2015; Hines-Beard et al., 2016). Neuroinflammation can, directly or indirectly, increase the activity of a number of kinases responsible for the phosphorylation of NF and tau in chronic neurodegenerations (For example, CDK-5: Kitazawa et al., 2005; Weston and Davis, 2007; Noda et al., 2014). Oxidative stress is also seen in glaucoma and other neurodegenerations (Chrysostomou et al., 2013; Inman et al., 2013; Burté et al., 2014; Kim et al., 2015; Yang et al., 2016) and reactive oxygen species such as peroxynitrate activate kinases including GSK-3β and p38 that modify tau and other proteins (Zhang et al., 2006). In addition, accumulation of Aβ_42_ and tau have been shown to lead to mitochondrial dysfunction and generation of reactive oxygen species (Luque-Contreras et al., 2014), effectively perpetuating the cycle of pathological phosphorylation and diminished axonal stability and function.

### Conclusions

In conclusion, data from the DBA/2J model of glaucoma demonstrate that increased phosphorylation of cytoskeletal proteins such as neurofilament-heavy and tau occurs with age and pathology within the RGC projection. However, the relationship is non-monotonic and overall loss of axonal structure with advanced pathology contributes to decreases in protein concentration at late glaucomatous ages. Although the presumptive, initial stressor (elevated IOP) occurs proximally at the optic nerve head, early manifestations of pathologies can occur distally, as previously reported (Conforti et al., 2007; Crish et al., 2010; Crish and Calkins, 2011) and are not isolated to the eye and retina (Parrish, 2003; Ito et al., 2012). Additionally, our data describing early, pre-degenerative elevations in Aβ_42_ and SBDP, have suggested that there are factors other than elevated IOP perpetuating glaucomatous neurodegeneration or predisposing RGC populations to respond adversely to high IOP later in life in the DBA/2J mouse.

These experiments provide new protein targets affected at pre- and early glaucomatous stages that may provide useful therapeutic tools for diagnosis or interventions that target more than IOP. Importantly, aberrant hyperphosphorylation of cytoskeletal elements, tau, and NF-H, occur prior to complete loss of anterograde transport and axon loss—supporting the idea that a therapeutic window exists in which subtle structural changes may be targeted to potentially prevent loss of function or structure. This also suggests that activity of GSK-3β and other kinases that lead to hyperphosphorylation and protein translocation/mislocation, along with calpain-induced spectrin breakdown, are early events with clear mechanisms for inhibition. Elucidating the timeline and efficacy of drugs affecting these pathways, therefore, is a promising avenue for future research aimed at slowing, stopping, or reversing early pathology and the ultimate neurodegeneration that produces vision loss in glaucoma.

## Author contributions

GW conducted all protein quantification assays, some immunofluorescence, and was involved in the design, analysis and interpretation of data as well as writing and revising the manuscript. MS assisted in sample collection, immunofluorescence and manuscript preparation. SC and DI conceptualized and designed the studies and assisted in the critical revision of the manuscript; CD assisted in immunofluorescent assays, analysis and interpretation of the data, as well as manuscript preparation and critically revising the manuscript.

## Funding

This work was supported by a grant from the National Eye Institute (EY022358) to SC and by a Young Investigator Fellowship from Prevent Blindness Ohio to GW.

### Conflict of interest statement

The authors declare that the research was conducted in the absence of any commercial or financial relationships that could be construed as a potential conflict of interest.

## Abbreviations

pNF-H: hyperphosphorylated heavy-chain neurofilament
SBDP: spectrin breakdown product
ON: optic nerve
SC: superior colliculus
GSK: glycogen synthase kinase
RGC: retinal ganglion cell.
